# The Equipment Used in the SF_6_ Technique to Estimate Methane Emissions Has No Major Effect on Dairy Cow Behavior

**DOI:** 10.3389/fvets.2020.620810

**Published:** 2021-01-29

**Authors:** Fabiellen Cristina Pereira, Dayane Lemos Teixeira, Laura Ann Boyle, Luiz Carlos Pinheiro Machado Filho, Shaun Richard Owen Williams, Daniel Enriquez-Hidalgo

**Affiliations:** ^1^Laboratorio de Etología Aplicada, Universidade Federal de Santa Catarina, Florianópolis, Brazil; ^2^Facultad de Agronomía e Ingeniería Forestal, Pontificia Universidad Católica, Santiago, Chile; ^3^Instituto de Ciencias Agroalimentarias, Animales y Ambientales (ICA3), Universidad de O'Higgins, San Fernando, Chile; ^4^Animal Welfare Program, IRTA, Veïnat de Síes, Girona, Spain; ^5^Teagasc, Animal and Grassland Research and Innovation Centre, Moorepark, Fermoy, Ireland; ^6^Agriculture Victoria Research, Ellinbank, VIC, Australia; ^7^Bristol Veterinary School, University of Bristol, Langford Campus, Langford, United Kingdom; ^8^Sustainable Agriculture Sciences Department, Rothamsted Research, Okehampton, United Kingdom

**Keywords:** cattle, enteric methane, milk production, lying duration, ruminating, habituation

## Abstract

The natural behavior of animals can be disrupted by the techniques and materials of research methodologies. This study aimed to evaluate the effect of the equipment used in the SF_6_ tracer technique to estimate enteric methane emissions on the behavior of lactating dairy cows. The cows (*n* = 24) were allocated to one of two diets: CONTROL and experimental diet (MIX). Behavior was assessed through video recordings between milking times during four phases: 3 days before fitting the cows with the SF_6_ equipment (PRE), first 2 days after the cows were fitted with the SF_6_ equipment (ADAP), 3 days during methane emission measurements (MEAS), and 2 days after the SF_6_ equipment removal (POST). The behaviors recorded included eating, ruminating or idling, resting, and others. Affiliative or agonistic and discomfort behaviors (scratching or pushing the equipment) were also recorded. Lying time was recorded over 14 days using dataloggers fitted to the cows' leg. Milk production and feed intake were recorded daily. MIX cows ruminated more than CONTROL cows (*P* = 0.05). The cows ruminated more at MEAS than in any other phase (*P* < 0.01). Time spent idling gradually decreased from PRE to MEAS for MIX cows (*P* < 0.01). The cows were lying down longer in MEAS than in ADAP and POST (*P* < 0.01). The time spent lying with the head down was shorter during PRE and ADAP than during POST (*P* < 0.05). No difference was observed in the occurrence of discomfort or agonistic behaviors (*P* > 0.05). Affiliative behaviors occurred more often in ADAP than in MEAS (*P* < 0.05). There was no difference between phases in daily lying time, number of lying bouts per day, or mean bout duration (*P* > 0.05). Milk production was not influenced by the SF_6_ equipment (*P* > 0.05). Dry matter intake was higher for CONTROL cows (*P* < 0.01), and it decreased from PRE to MEAS (*P* < 0.01). However, milk yield did not differ between cows wearing the SF_6_ equipment and those without it (*P* > 0.05). We conclude that the SF_6_ equipment had a minimal effect on dairy cow behavior.

## Introduction

A requirement of research is the control of variables that are not under investigation. In animal research, this implies that experimental animals should be able to behave normally (1). Changes in animal behavior are the first visible reaction to a particular stimulus in an animal's environment (2), and changes in the animal's behavior provide information about its physiological and psychological state (3). Behavioral changes do not always occur in an adaptive and beneficial way (2). In some cases, they may indicate health problems, emotional disturbance, and stress (4) and may influence animal productivity (5).

For dairy cows, the disruption of natural behavior related to environmental and social conditions in which they are maintained is well documented. Ingestive behaviors, for instance, can be affected either by changes in the social environment (6) or in feed availability and its characteristics (7), both of which can influence feed intake and rumination duration. Similarly, changes in lying behavior can indicate cow discomfort and welfare issues (8), with consequent adverse effects on milk production (9).

Natural behaviors can also be disrupted by the techniques and materials of research methodologies (10). An example of this is the technique used for measuring brain activity to describe stages of sleep in cows (11, 12). Besides the equipment used, such methods usually require management routines during the measurements, which can modify their behavioral patterns and the behaviors being measured by the technique as well (13). However, there is a shortage of information regarding the effect of such specific practices on dairy cow behavior. Many researchers validate equipment used to measure cow behavior through comparison of the results by direct visual observations vs. the simultaneous measurements made by the equipment (14, 15) rather than testing the effects of its use *per se*. Alternatively, previous studies compared the data provided by different types of equipment without comparing the behavior of the cows when they are using particular equipment to when they are not (13, 16).

Dairy cows are usually able to change their behavior to adapt to new routines or conditions (17) without detrimental effects on their health, welfare, or productivity. For this purpose, a period of habituation or adaptation is required before beginning an experiment. Where cows are unable to adapt to the research conditions, even after this period, the research outcome may be compromised, leading to biased or irrelevant results.

Misleading results can become a problem when data are used to produce national reports, such as greenhouse gas inventories. Studies that are used to produce this kind of report must be consistent and offer reliable and accurate data based upon realistic and reliable real-life statistics (18, 19). Several methods can be used to estimate the gases emitted from cattle; these are based on a variety of equipment, laboratory techniques, tracers, sensors, and mathematical models (20).

The respiration chamber method is the standard method used to measure the emission of enteric methane (CH_4_) from ruminants (20). However, the need to keep the cows enclosed to collect the data (21) is criticized for restricting natural behavior patterns (grazing, walking, interaction with other animals, etc.), thus potentially generating erroneous data with a high coefficient of variation (22). One reason that the sulfur hexafluoride (SF_6_) tracer method was developed was to enable the estimation of CH_4_ emissions from individual ruminants in their natural environment (23). The SF_6_ method was validated to estimate CH_4_ emissions, and adaptations of the technique were made to make the equipment more comfortable for the cows (24). However, to our knowledge, there is no information regarding the effect of such equipment on the behavior of dairy cows. As the SF_6_ technique involves fitting the cows with a saddle and a halter for a short period of time (i.e., 1 week), it has potential to alter their behavior. We hypothesized that cows would show an ephemeral resistance to the equipment, manifested by changes in behavior that could potentially influence their performance and ultimately affect CH_4_ emission measurement. The frequent and close contact with the animals required by this technique might also be a problem if they are not used to the presence of humans or handling (20). A short period of habituation might not be enough to habituate cows to the equipment and daily handling. Therefore, the objective of this study was to evaluate the effect of using the SF_6_ technique to estimate CH_4_ emissions on dairy cow behavior over a short-term period.

## Materials and Methods

The experiment was conducted at the experimental station of the Pontificia Universidad Católica de Chile, located 25 km south of Santiago of Chile, in Pirque, 33°40′ south and 70°36′ west, from August to October 2018. The study was approved by the Scientific Ethics Committee for Animals and Environmental Care of the Pontificia Universidad Católica de Chile (protocol number 160511004).

### Animals and Experimental Description

Twenty-four lactating dairy cows from a herd of 220 cows split into two groups according to their number of days in lactation (days in milk, DIM) (0–200 or >200 DIM) were selected and assigned to one of 12 blocks according to genotype (Holstein–Friesian and Montbeliard), lactation number (1.6, SD 0.76), DIM (222d, SD 84.7), and pre-experimental milk yield (37.8 kg/day, SD 4.27). The cows within blocks were randomly allocated to one of two groups to evaluate the effect of two experimental diets on CH_4_ emissions [described in (25)]. Briefly, both groups were separated from the main herd for 10 weeks and randomly allocated in individual pens (6.0 × 3.5 m) separated by wooden slat fences, which allowed interaction with neighboring cows. The pens were cleaned twice a day, and the sawdust bedding material was changed daily. Each base group received one of two different iso-energetic and iso-proteic experimental diets offered as total mixed rations that differed in the type of base forage used over a 10-week period: a CONTROL diet similar to the diet usually offered to the cows where the forage fraction was made of corn silage (*Zea mays* L.) and alfalfa (*Medicago sativa* L.) hay and an experimental diet (MIX) where a fresh annual ryegrass (*Lolium multiflorum* L.) and berseem clover (*Trifolium alexandrinum* L.) mixed herbage was used to partially replace the corn silage and alfalfa hay forage fraction of the CONTROL diet. The composition of the diets are shown in Table 1. The cows had *ad libitum* access to their diets (regulated to 5% refusals) and to an individual water trough. The amount of feed offered to each cow and their individual residuals were weighed daily to estimate the average dry matter intake (DMI). The cows were milked three times per day at 8:00, 15:00, and 21:00 h, and the diet was offered twice a day, after the morning and the afternoon milking, respectively. The effect of the diets on productive variables and individual CH_4_ emissions are available in Enriquez-Hidalgo et al. (25).

**Table 1:** Configuration [% of dry matter (DM) basis] of the control (CONTROL) and experimental (MIX) diets offered to the cows during the experiment [adapted from (25)].

**(% of DM)**	**CONTROL**	**MIX**
Ground corn	16.7	20.2
Wheat bran	14.4	18.2
Canola	23	11.2
Maize Silage	34.2	25.2
Alfalfa hay	8.4	–
Ryegrass and berseem clover	–	25.2

### The Sulfur Hexafluoride Methane Emission Equipment

**Figure 1:**
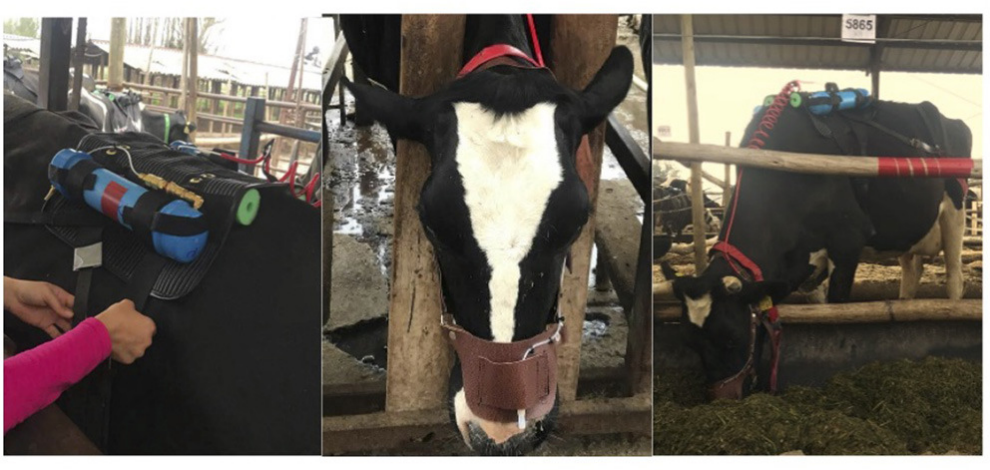
Cow equipped with the sulfur hexafluoride (SF_6_) equipment to measure enteric methane (CH_4_) emissions. From left to right: the saddle with PVC canisters; leader head halter, with the sampling point; and the entire equipment.

The individual CH_4_ emissions were estimated after the cows have been in individual pens for 6 weeks. Due to equipment availability, the emission measurements were undertaken on 2 weeks split over two groups of cows. The emissions of the first 12 cows (from six blocks: six cows from each diet treatment) were estimated on week 7 and of the second group of 12 cows (from the other six blocks: six cows from each diet treatment) on week 9 of the experiment. We used the modified SF_6_ technique (24) to estimate CH_4_ emissions. Briefly, the equipment consisted of a leather head halter, with the sampling point above the nostrils (Figure 1). Two sample collection PVC canisters were mounted on a padded, flexible saddle that was fitted to the cow with a foam-padded girth-strap and a plastic strap placed around the cow's hindquarters. A complete set of equipment weighed approximately 3.3 ± 0.05 (SD) kg. At the specific weeks, the animals were fitted with the SF_6_ equipment, and the equipment remained on the cows for seven consecutive days. Every day, after the morning milking, the cows had the PVC gas-sampling canisters replaced, and all checks of the function of equipment and necessary adjustments were undertaken as required by the technique (24). The time that the cows spent outside their pens due to milking, equipment adjustment, and changing of the canisters was recorded on a daily basis during the experiment.

### Measurements

#### Behavioral Evaluations

The experiment evaluated cow behavioral changes before and after their exposure to a new condition following a similar layout as that of Enriquez-Hidalgo et al. (26). The evaluations were carried out at four different phases:

1. three consecutive days before the cows were fitted with the SF_6_ equipment (PRE),
2. first 2 days while the cows were fitted with the SF_6_ equipment, in which, firstly, the saddle was put on and adjusted and, secondly, the halter and canisters were added (ADAP),
3. three consecutive days during the CH_4_ measurements (MEAS), and
4. two consecutive days after the SF_6_ equipment was removed (POST).

The cow behavior between milking sessions (10:00–14:00 and 16:00–20:00) was video-recorded (H.264 network digital video recorder; SECO-LARM; Enforcer DR-1, Irvine, CA, USA). We employed instantaneous scan sampling at 10-min intervals to measure cow behavior, yielding 480 observations per cow. The following behaviors were recorded: ingestive behavior (eating, ruminating, or idling), resting behavior (lying with the head up or lying with the head down), and others (grooming and any other behavior different from the behaviors described above). At each specific minute, the person watching the videos record the instantaneous behavior being performed by each cow based on the detailed descriptions of each behavior presented in Table 2. Then, during the last 60-s period of every 10 min of observation, all occurrences of discomfort behaviors (scratching, pushing, or licking the equipment—halter or saddle) and social interactions (affiliative or antagonistic) were counted and recorded (27). The same person watched all the videos and recorded all the behaviors to avoid different interpretations.

**Table 2:** List and description of the evaluated behaviors.

**Behavior**		**Description**
Ingestive	Eating	Picking or consuming food, with the head close to the ground in the feeder
	Ruminating	Chewing regurgitated boluses of feed
	Idling	Standing, no ruminating
Resting	Head up	Lying in a rest position with the head lifted
	Head down	Lying in a rest position with the head on the soil
Other	Grooming	Licking itself
Discomfort	Scratching	Scratching the equipment against the wooden fence
	Pushing	Pushing the equipment with the head
	Licking	Licking the equipment or the body area around it
Social interactions	Affiliative	Licking the neighbor cow
	Agonistics	Head butting, pushing, threatening, and/or fighting with the neighbor cow

#### Daily Lying Behavior as Recorded by Dataloggers

Lying behavior, number, and duration of lying bouts per day were recorded over 14 consecutive days, coinciding with the days of behavior observations. Dataloggers (HOBO dataloggers Pendant G Acceleration—Onset Computer Corporation) were fitted below the hock on the outside of the right hind leg or on the inside of the left hind leg of each cow and secured using a Vetwrap™ bandage as per O'Driscoll et al. (28). They were set to record whether the cow was standing or lying at 5-min intervals and also the number and duration of lying bouts per day using HOBOware Lite (Onset Computer Corporation, Bourne, Massachusetts, USA).

#### Dry Matter Intake and Milk Yield

Individual DMI and milk yield were measured daily throughout the experimental period, but not during the ADAP phase; therefore, data were combined and evaluated in two different ways: (1) during PRE, MEAS, and POST phases following the same methodology as the behavioral observations and (2) comparing the data from the cows wearing the equipment with those that were not wearing it during the CH_4_ measurements (MEAS).

### Statistical Analysis

All behavioral data were summarized as one value per cow per day, while milk yield and DMI were summarized as one value per cow per phase or stage. All analyses were undertaken using generalized linear mixed models (Proc Glimmix) in SAS (SAS Institute Inc., Cary, North Carolina, USA). Normality and homogeneity of variances were checked by analysis of the residuals, and the distribution (normal, gamma, and Poisson) was defined according to its visual evaluation. Least square differences were evaluated using the Tukey test adjustments for multiple comparisons, considering significant differences at *P* < 0.05. The model considered the phases, the diets, and the phase × diet as fixed effects and cow within block as random effect. The phase × diet effects were removed from the models if they did not tend (*P* > 0.1) to influence the variable response. The day nested within phase was used as repeated measurement, and the cow was used as experimental unit. Milk yield and DMI were analyzed using two different approaches: (1) submitted to a similar model as for the behavioral data but the stage was used instead of the phase and (2) a model considering whether the cows were fitted or not with the SF_6_ equipment and the diet effects [the interaction between these variables was initially tested and then removed from the final model as it did not tend (*P* > 0.1) to influence the variable response]. The results are presented as least square mean ± standard error of the mean (SEM). Individual datalogger data that were <20 and >70% of the daily time lying were considered as equipment errors and were not included in the analysis (overall, three cow days of data were excluded from the analysis).

## Results

On average, during PRE and POST phases, the cows remained in each milking time at around 1.7 (0.64) and 2.6 (0.40) h while in ADAP phase due to required adjustments and 1.8 (0.57) h when in MEAS phase due to daily changing of the canisters and any equipment that required adjustments.

The diet and the phase × diet interaction did not (*P* > 0.1) influence most of the analyzed variables; therefore, these effects were removed from the models, and there are no further comments regarding these effects unless otherwise noted.

### Behavioral Evaluations

Table 3 shows the duration and frequency of behaviors in each phase. We observed no difference in percentage of the day engaged in eating behavior. The greatest percentage of the day spent ruminating was observed in MEAS (*P* < 0.02) and the lowest in PRE (*P* < 0.05). The cows that received the MIX diet tended to ruminate more than the cows that received the CONTROL diet (22.8 vs. 19.9; SEM, 1.06%; *P* = 0.051).

**Table 3:** Cow behavior in each of four phases of fitting with sulfur hexafluoride (SF_6_) equipment.

**Behavior**	**PRE**	**ADAP**	**MEAS**	**POST**	**SEM**	***P*-value**
**Time proportion (%)**						
Eating	35.5	36.1	35.8	36.9	1.74	0.94
Ruminating	17.9^b^	20.9^b^^c^	25.2^a^	21.4^c^	1.12	<0.001
Idling	38.2^a^	32.9^b^	31.0^b^	33.7^b^	2.27	<0.01
Resting head up	42.6^b^	40.7^b^	47.0^a^	39.4^b^	2.27	<0.01
Resting head down	5.6^b^	5.1^b^	6.1^a^^b^	8.8^a^	0.75	0.01
Other	5.7	6.5	5.7	6.7	0.42	0.17
**Activity frequency (No./h)**						
Halter interactions	–	0.04	0.19	–	0.05	0.06
Saddle interactions	–	0.12	0.10	–	0.05	0.93
Grooming	0.81	0.72	0.74	0.88	0.13	0.87
Agonistic	0.31	0.51	0.40	0.61	0.15	0.77
Affiliative	1.28^a^^b^	1.65^a^	0.84^b^	0.88^a^^b^	0.3	0.02

a−c *Means within a row with different superscripts differ (P < 0.05)*.

*SEM, standard error of the mean; No., number*.

*PRE, Before cows had the equipment on; ADAP, initial period of fitting; MEAS, During the enteric methane measurements; POST, after the SF_6_ equipment was removed*.

The percentage of the day that the cows spent idling decreased (*P* < 0.01) when the cows were fitted with the SF_6_ equipment during ADAP phase, which was maintained until POST phase. However, there was an interaction between phase and diet (*P* = 0.05). The cows that received the MIX diet gradually reduced the time spent idling from PRE to MEAS phase (PRE: 37.5, ADAP: 29.1, MEAS: 27.3; SEM, 3.15%; *P* = 0.01). We did not observe any difference between phases for the cows that received the CONTROL diet (33.2 ± 2.85%; *P* = 0.55).

The percentage of the day that the cows were resting with their head up was greatest (*P* = 0.02) during the MEAS phase. Meanwhile, the cows were lying with their head down more (*P* < 0.05) after the use of the equipment (POST) than in PRE and ADAP, but it was similar to MEAS.

We observed no difference in the percentage of the day engaged in “other” behaviors or interaction with the equipment (halter or saddle) between the different phases.

There was no difference in agonistic interactions between phases, yet affiliative behaviors slightly increased at ADAP and decreased during MEAS (*P* < 0.05 for both).

### Daily Lying Behavior as Recorded by Dataloggers

**Figure 2:**
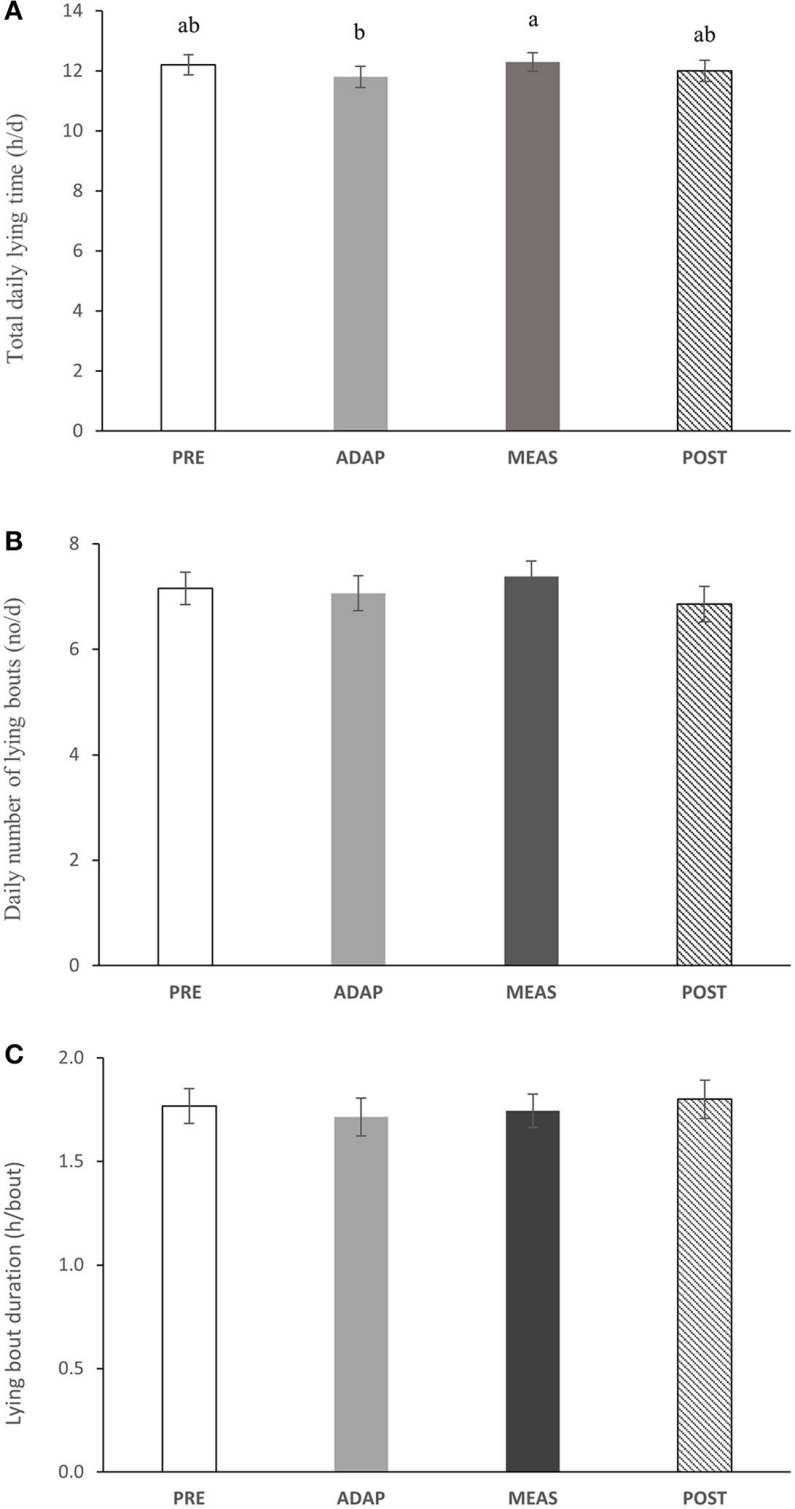
Effect on behavior of cows wearing sulfur hexafluoride (SF_6_) equipment to measure enteric methane (CH_4_) emissions in each of four phases [PRE, Before cows had the equipment on; ADAP, initial period of fitting; MEAS, During the enteric methane (CH_4_) measurements; POST, after the SF_6_ equipment was removed] on **(A)** total daily lying time (h/d), **(B)** daily number of lying bouts (no/d) and **(C)** lying bout duration (h/bout).

Total lying time tended (*P* = 0.06) to change during phases, with the cows spending 4.1% (*P* = 0.05) less time lying while in the ADAP phase than in the MEAS phase (Figure 2A), but no other differences were detected. We noted no difference in the number of lying bouts per day (7.1 ± 0.31 times/day, *P* = 0.74; Figure 2B) or in mean bout duration (5.2 ± 0.08 h/day, *P* = 0.22; Figure 2C).

### Dry Matter Intake and Milk Yield

The cows eating the CONTROL diet had greater DMI than MIX cows (24.1 vs. 21.1; SEM, 1.60 kg/day; *P* = 0.004). DMI decreased from PRE to MEAS phase (*P* = 0.004) and had an intermediate value in POST (Table 4). However, the cows fitted or not with the equipment had similar DMI (Table 5). The CONTROL cows produced more milk per day than the cows that ate the MIX diet (34.7 vs. 28.9; SEM, 1.20 kg/day; *P* < 0.001), but milk yield was the same throughout the different phases (32.4 ± 1.28 kg/day, *P* = 0.44; Table 4). There was no difference in milk yield between the group of cows wearing the SF6 equipment and those without it (Table 5).

**Table 4:** Milk yield and dry matter intake of dairy cows in three phases of fitting with sulfur hexafluoride (SF_6_) equipment.

	**PRE**	**MEAS**	**POST**	**SEM**	***P*-value**
Milk yield (kg/day)	32.2	32.2	31.0	1.28	0.44
Dry matter intake (kg dry matter/d)	29.2^a^	23.0^b^	26.0^a^^b^	1.74	<0.01

a−b *Means within a row with different superscript letters differ (P < 0.05)*.

*SEM, standard error of the mean; PRE, before the cows had the equipment on; MEAS, during the enteric methane measurements; POST, after the SF_6_ equipment was removed*.

**Table 5:** Milk yield and dry matter intake of dairy cows fitted or not with sulfur hexafluoride (SF_6_) equipment.

	**Cows fitted with SF_**6**_ equipment**	**Cows not fitted with SF_**6**_ equipment**	**SEM**	***P*-value**
Milk yield (kg/day)	32.2	32.1	1.23	0.96
Dry matter intake (kg dry matter/day)	23.1	22.0	1.69	0.56

*SEM, standard error of the mean*.

## Discussion

Fitting the cows with the SF_6_ equipment did not change their behavior. The short period of habituation was sufficient to allow the cows to become accustomed to the equipment before the data collection period commenced.

Lying behavior is a priority behavior for cows and a strong indicator of their comfort and welfare (29). Where changes in cow lying behavior are noted, it can indicate that the animals are stressed (3). In this sense, lying behavior is widely used to analyze the cows' ability to cope with a specific environment or management practices (7). We detected no difference in lying behavior between the phases evaluated, except for a shorter lying time during the ADAP phase compared to the MEAS phase. This could be related to the introduction of the cows to the equipment, followed by their habituation, as reflected in an increase in time spent lying back to PRE levels. Such a change is more subtle than that observed by Enriquez-Hidalgo et al. (26) when evaluating changes in lying behavior during the transfer of dairy cows from pasture to tie-stalls. They reported indicators of discomfort due to the transfer, but this disappeared after some days of adaptation as reflected in fewer but longer lying bouts. We did not note a difference in lying bouts or bout duration though. In both cases, this reluctance is expected since the cows are presented to something unusual from their routine. Nevertheless, as soon as they were familiar with the new condition they were submitted to, they were able to execute their basic activities normally. Contrary to the effect presented here and with the results of Enriquez-Hidalgo et al. (26), Johns et al. (30) reported that cows did not habituate to the use of bells, even though they had previous experience to bells, since lying time and lying bout duration were decreasing even more after some days. These contrary results highlight the fact that cows are able to adapt to something unknown as long as it is not stressful to them. Otherwise, a period of habituation might not make a difference as had occurred with the bells in Johns et al. (30). In our work, it is likely that the minor effect of the SF_6_ equipment on the cows' lying behavior was overcome by the cows during the phase of habituation.

The time spent ruminating was affected by the phases and diets, which was in line with expectations given the sensitivity of rumination to food characteristics (31), environmental stressors (15), and unusual handling (30). This can affect feed intake and thereby be associated with welfare issues (15). Johns et al. (30) found that the cows spent less time ruminating and eating when either functional or silent bells were used, indicating stress due to the weight and also the sound of the object. However, in our study, ruminating time increased during the use of the SF_6_ equipment (MEAS), and no difference in eating behavior was noted. We did notice a reduction in DMI during the MEAS phase, but when comparing the cows fitted and the cows not fitted with the equipment during the measuring period, feed intake was the same. Therefore, we speculate that the increase in ruminating time might be due to other factors that we did not measure. Management routine such as feeding, milking, and cleaning can also affect ruminating time (13). Overall, the daily activities followed the normal routine for the farm. However, small differences in feeding time or milking duration might have also influenced ruminating time. In addition, the fact that the behaviors were not assessed during nighttime, when ruminating occurs more frequently, may have influenced our results. Another possible explanation could be due to the longer time that the cows spent resting with their head up during the MEAS phase since there is a positive relationship between ruminating and lying behavior, and the cows show a preference for ruminating while lying down (32). The cows that received the MIX diet tended to spend more time ruminating than the cows that received the CONTROL diet. We can associate this with the inclusion of fresh forage. The fresh forage was expected to increase the fiber content and the particle size of the diet, thus increasing the resistance to chewing and the rumination time (31). Moreover, there was a 75% increase in the herbage mass of the mixed herbage and a more advanced maturity stage for the ryegrass in the swards during the methane emission measurement week of the second group (25), but no apparent increase in the MIX diet fiber concentration was noted. The use of the SF_6_ equipment had no negative effect on ruminating behavior. Indeed given the fact that ruminating activity is an important indicator of cow welfare (33), the increase in this behavior shows us that the cows were comfortable.

All the cows gradually decreased their time spent idling from ADAP phase to POST phase, but this reduction was more accentuated for cows eating the MIX diet. The time spent idling is normally the inverse of the time dedicated for eating or ruminating (34). In this way, once ruminating time increased on the ADAP and MEAS phases, we can expect to see a reduction in idling. The feed composition can also influence the time spent idling (34), which, therefore, explains the difference in this behavior between the two diets.

The cows mostly rested with their heads up. The position of the head during resting behavior is associated with different states of vigilance in cows (12). Resting with the head on the ground, associated with rapid eye movement, indicates a deep stage of sleep (35). According to Ruckebusch (11), this state of sleep totals about 45 min of the cow's day, while about 3 h per day is the total of the non-rapid eye movement, a position in which the head is lifted from the ground and supported by the neck (12). Both states of sleep are higher during nighttime (16). Although we did not evaluate the behaviors during the night, our observations are in accordance with those of Ruckebusch (11) regarding the proportion of time with the head in both up and down positions. Rapid eye movement sleep is a priority to cows and can be reduced in stressful situations (35). The time that the cows spent resting with their heads down was higher during the MEAS and POST phases; therefore, it seems that the cows were comfortable enough to rest even when wearing the equipment. Changes in the cows' environment and handling can cause disturbances in the distribution of the different stages of sleep (12). This could mean that the stress of handling influenced resting rather than it being associated with equipment use. These results, combined with the lack of effect of the interaction between the cows and the equipment (halter or saddle) during the phases, indicate that the cows were comfortable while fitted with the SF_6_ equipment.

Social interactions did not differ between stages. Only affiliative interactions, noticed mainly as mutual grooming on the upper part of their bodies, changed according to the phases, increasing during the ADAP phase and decreasing during MEAS. In spite of being poorly understood and neglected because of its difficulty of measure and definition, social support is linked to the ability of animals to cope with stressful or challenging situations (36). Taking into account that the equipment was something new for the cows, we can consider it as a challenging factor to them during the ADAP phase, so it may have stimulated the cows to come closer to their neighbors. Ishiwata et al. (37) noticed the preference of cattle to group together with other familiar heifers in order to mitigate handling stress. Laister et al. (38) concluded that social grooming has a calming effect in cows, having observed reductions in the heart rate of cows while receiving licking as a sign of social grooming. The SF_6_ equipment may not have been a stressor to the cows but simply something unusual from their normal environment. As soon as the cows were familiar with it, their degree of fearfulness decreased (39).

Stress may negatively influence cow performance (5, 40). In this study, there was no difference in milk yield between the phases and no effect of treatment during MEAS phase. Dry matter intake decreased from PRE to MEAS phase; nevertheless, it was the same for all cows. Therefore, another factor influenced all the cows to reduce their DMI rather than the use of the equipment, which allows us to assume that wearing the equipment has no physiological effect as to impact production indicators. Both milk yield and DMI though were reduced in cows that were offered the MIX diet, which might be related to differences in nutritional composition between diets.

Despite that, our results suggest that the SF_6_ equipment has no major influence on the behavior of dairy cows. Our study was undertaken with the cows allocated in individual pens. Thus, it is possible that cows in free-range conditions may respond differently to the use of the SF_6_ equipment, and this aspect warrants further investigation.

## Conclusion

We conclude that the SF_6_ equipment has no major influence on the behavior of dairy cows. We observed only minor changes associated with the introduction of the cows to the equipment. Hence, the SF_6_ technique can be used with confidence since it should not interfere with cow behavior in a way that would affect productivity and, thus, methane emission outcomes.

## Data Availability Statement

The original contributions presented in the study are included in the article/supplementary materials, further inquiries can be directed to the corresponding author/s.

## Ethics Statement

The animal study was reviewed and approved by Scientific Ethics Committee for Animals and Environmental Care of the Pontificia Universidad Católica de Chile (protocol number 160511004).

## Author Contributions

DE-H, DT, and FP contributed to the concept of this work and designed the study. DE-H and DT performed the statistical analysis. FP and DE-H performed the experiment and wrote the manuscript. DT, LP, LB, and SW contributed to the manuscript. All authors helped with data interpretation and approved the final version of the manuscript.

## Conflict of Interest

The authors declare that the research was conducted in the absence of any commercial or financial relationships that could be construed as a potential conflict of interest.
